# Clinico-microbiological Profile of Nontuberculous Mycobacterial Keratitis

**DOI:** 10.18502/jovr.v17i2.10786

**Published:** 2022-04-29

**Authors:** Richa Dhiman, Meena Lakshmipathy, Dhanurekha Lakshmipathy, Therese K.Lily

**Affiliations:** ^1^CJ Shah Cornea Services, Medical Research Foundation, Sankara Nethralaya, Chennai, Tamil Nadu, India; ^2^L & T Microbiology Research Center, Vision Research Foundation, Sankara Nethralaya, Chennai, Tamil Nadu, India

**Keywords:** Atypical Mycobacterial Keratitis, M. Abscessus, Nontuberculous Mycobacterial Keratitis, Polymerase Chain Reaction

## Abstract

**Purpose:**

To assess the clinical and microbiological characteristics of nontuberculous mycobacterial (NTM) keratitis and to evaluate their response to medical therapy.

**Methods:**

Sixteen patients of NTM keratitis were retrospectively reviewed from May 2014 to May 2019. Laboratory diagnosis were made using Ziehl-Nielsen acid-fast staining, routine culture method of isolation of nontuberculous mycobacteria and further identification of species by PCR (polymerase chain reaction)-based DNA sequencing targeting the heat shock protein-65 (*hsp-65*) gene.

**Results:**

Sixteen patients of microbiologically proven NTM keratitis were included. The average age at the time of presentation was 43.56 years (range, 24–73 years). The mean duration of symptoms was 2.23 months. The commonest risk factor was injury with organic material (43.7) followed by ocular surgery (25%). The majority of the nontuberculous mycobacteria were *Mycobacterium abscessus* (87.6%) followed by *M. fortuitum *(6.2%) and *M. chelonae *(6.2%). The *in vitro* sensitivity showed maximum sensitivity to Amikacin (AMK; 100%) followed by Azithromycin (AZM; 85.7%), and Clarithromycin (CLR; 85.7%). Out of a total of 16 patients, 12 (75%) had total success with medical therapy while 4 (25%) required surgical intervention.

**Conclusion:**

This study is focused on rapid and reliable identification of NTM keratitis through PCR-based identification method to enable effective medical management. The antibiotic susceptibility testing of different subspecies of NTM further reduced the need for surgical intervention. The effective role of AMK either alone or in combination with macrolide antibiotics is also highlighted in this study.

##  INTRODUCTION

Nontuberculous mycobacteria (NTM) also known as atypical mycobacteria are free-living, aerobic, non-sporulating, and non-motile bacilli. Although they are ubiquitous, for the past two decades, their frequency has increased in surgical instruments and surgical suites, especially after refractive procedures.^[1,2,3]^ They have the ability to survive in artificial environments like daily water distribution systems, pools, and operating rooms on account of their significant pathogenic property of biofilm formation which shields the NTM from disinfectants.^[4]^ In 1965, the first case of chronic keratitis was reported by Turner et alwhich was caused by* Mycobacterium fortuitum*, following the removal of a corneal foreign body.^[5]^ Out of the total cases of NTM keratitis, the *M. fortuitum *group and the* M.* *chelonae *(*M. chelonae – M. abscessus*) groups are responsible for 83.5% of the cases while the Runyon groups I–III, the slowly growing mycobacteria (SGM), accounts for other 16.5% of the cases.^[6]^ Several risk factors like history of ocular trauma, use of contact lenses, ocular implants, steroids, and multiple surgeries have been related with NTM ocular infections. These infections are a challenge to diagnose clinically and microbiologically leading to delay in the diagnosis and treatment. They have an indolent course that is prolonged if topical steroids are used. The NTM show varying degrees of susceptibility to the commonly used antibiotics including aminoglycosides, fluroquinolones, and erythromycin. The association of *in vitro* susceptibility and clinical response is not strong and surgical intervention is frequently required to eradicate the intractable infections caused by NTM. Hence, NTM species identification by molecular techniques play a crucial role in the management of NTM keratitis.

This study was done to assess the clinical and microbiological characteristics of eyes with NTM keratitis and evaluate their response to medical therapy and their clinical outcome with special emphasis on early diagnosis by PCR-based DNA sequencing targeting *hsp65* gene for definite species recognition.

##  METHODS

We retrospectively reviewed 2759 cases of microbial keratitis from May 2014 to May 2019 after appropriate approval from the institutional review board adhering to the tenets of the Declaration of Helsinki. Of these, 16 cases of NTM keratitis were identified. The study included a review of patients' records for the mode of presentation, clinical details, and outcome. Microbiological records of all the ocular specimens processed at the ocular microbiology laboratory were also reviewed.

The corneal scrapings from all 16 patients were taken from the base and edge of the ulcer using a sterile 15 no. blade after instilling local anesthetic (0.5% proparacaine) under slit-lamp magnification and then they were smeared on the glass slides and inoculated on to the routine culture media directly.

In most of the cases, the treatment was initiated based upon the direct smear report. Later, the treatment was modified as per the culture, antibiogram report from the laboratory, or if the clinical response to the medication was inadequate.

### Laboratory Procedures

#### Direct smear and culture

Smears were made for 10% KOH wet mount, Gram's stain and Acid-fast stain. The microorganisms with morphological features indicative of mycobacteria on Gram stain were then confirmed with Ziehl–Nielsen stain. The culture methods included in the study were as follows: Blood agar, Chocolate agar, Mac Conkey agar, Sabouraud's dextrose agar, and two liquid media – Thioglycolate broth and Brain heart infusion broth. Based on clinical suspicion, non-nutrient agar with *Escherichia coli* confluent overlay was included in the culture for isolation of Acanthamoeba.

NTM were identified by their typical colony morphology, rate of growth within 48–72 hr, and ability to grow on blood agar. If poorly staining gram-positive bacilli were seen on smears made from growth on blood agar, Ziehl–Nielsen stain was done to confirm the acid fastness of the bacterial growth. Simultaneously, all isolates were also subjected to PCR-based DNA sequencing method of species identification.

#### Extraction of DNA and PCR targeting hsp65 gene

DNA extraction was performed by using a Qiagen DNA extraction mini kit (Hilden, Germany) as per the manufacturer's instructions. For the PCR amplification, a 50 μl reaction was set with 5 μl of 10XPCR buffer (100 mM Tris-HCl [pH 8.3], 500 mM KCl, 0.1% gelatin, 15 mM MgCl2), 8 μl of 200 μM of each dNTPs, 1 μl of 1 picomole of each primer^[7]^ [Forward primer Tb 11: 5' ACCAACGATGGTGTGTCCAT 3', Reverse primer Tb12: 5' CTTGTCGAACCGCATACCCT 3'], 0.75 μl of 2 units of *Taq *polymerase, 5 μ l Sterile Glycerol, and 5 μl of extracted DNA was used as the template and the final volume was reached up to 50 μl with sterile Milli Q water. The positive control used for the experiment was *M. tuberculosis *H37Rv ATCC DNA.The PCR thermal profile consists of 40 cycles of denaturation at 94ºC for 1 min, annealing at 55ºC for 1 min and extension at 72ºC for 1 min and a final extension at 72ºC for 10 min. Amplification of the 439-bp product of the *hsp65* gene was identified by 2% agarose gel electrophoresis incorporated with 0.5 μg/ml ethidium bromide for visualization using a UV transilluminator.

**Figure 1:**
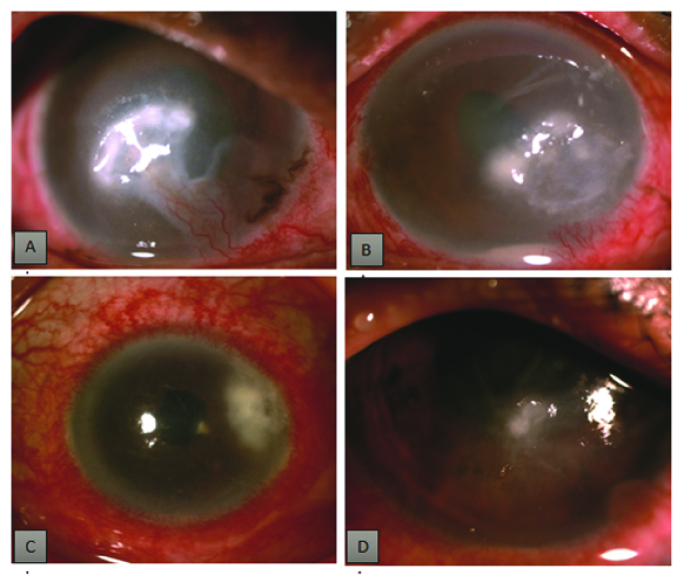
Nontubercular mycobacterial keratitis. (A) After trauma with vegetative matter showing infiltrate with vascularization. (B) Dry-looking infiltrate mimicking fungal infection with hypopyon which was diagnosed as NTM on repeat scraping. (C) Dense infiltrate after cataract surgery. (D) Paracentral infiltrate with severe stromal edema.

**Figure 2:**
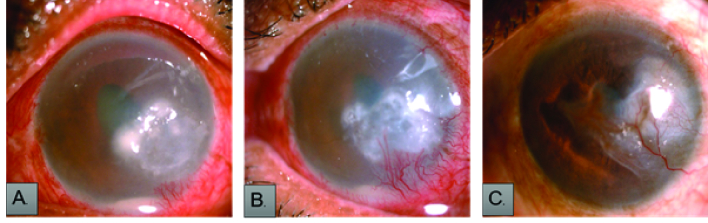
Slit-lamp image of caseno. 4 which was treated as fungal corneal ulcer elsewhere came positive on corneal scraping (Ziehl Neelsen stain) after repeating twice (A) at presentation; (B) 15 days posttreatment with topical fortified amikacin (2.5%) and oral clarithromycin; and (C) after two months, scarred infiltrate with corneal vascularization.

**Figure 3:**
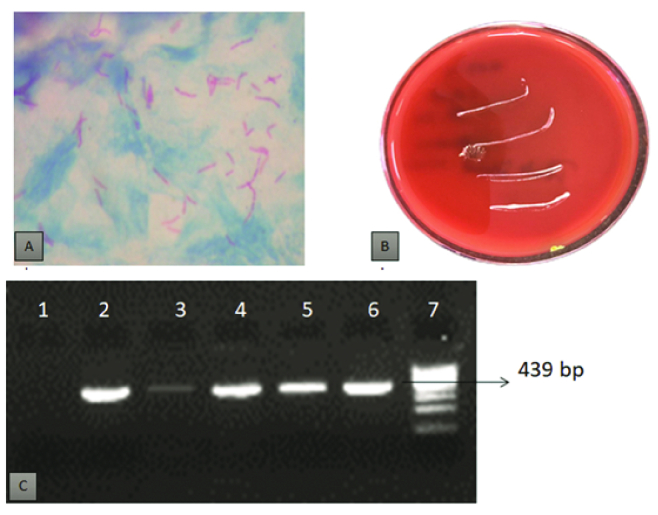
(A) Ziehl Neelsen-stained corneal scrapings showing acid-fast bacilli against a blue background. (B) White colored, opaque, non-hemolytic colonies of NTM on blood agar. (C) Amplification of a 439 bp-specific DNA fragment of the *hsp 65 *region of mycobacterial DNA. Lane 1: Negative control; Lane 2–5: Amplified mycobacterial DNA (439 bp); Lane 6: Positive control; and Lane 7: Molecular weight marker (100 bp).

**Figure 4:**
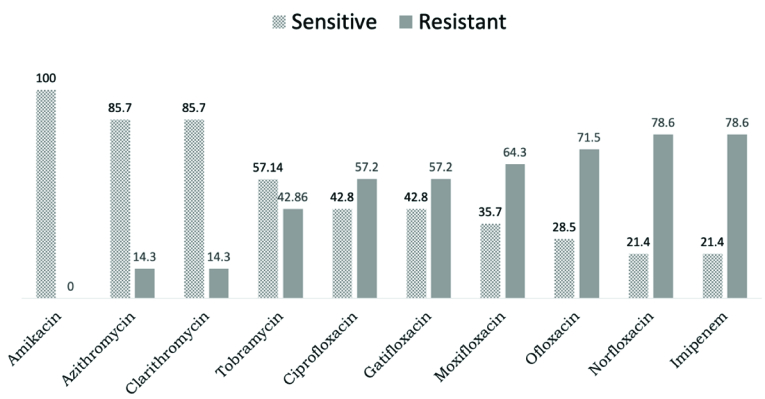
Bar diagram showing the sensitivity and resistance pattern of *in vitro* antibiotic susceptibility pattern of NTM isolates (*M. abscessus*) included in the study (*n* = 14).

**Table 1:** Clinical and microbiological details of 16 patients with NTM keratitis.


**No.**	**Age/Sex**	**Risk factors**	**Visual acuity at presentation (log MAR)**	**Depth of corneal infiltrate**	**Corneal scraping (AFB stain)**	**PCR (species)**	**Clinical outcome**	**Visual acuity at follow-up (log MAR)**	**Duration of follow-up**
**1**	38/M	Trauma	2	Full	Positive	*M. abscessus*	Failure	2.3	1 year
**2**	55/M	Nil	1.3	Anterior 2/3	Positive	*M. abscessus*	Total success	0.47	2 months
**3**	30/M	Steroid use	2	Ring	Positive*	*M. abscessus*	Failure	2	1 year
**4**	66/M	Trauma	0.47	Anterior 2/3	Positive*	*M. abscessus*	Total success	0.87	6 months
**5**	29/M	Foreign body	1.3	Anterior 1/3	Negative	*M. abscessus*	Total success	0.3	4 months
**6**	47/F	Nil	0.3	Anterior 2/3	Negative	*M. abscessus*	Total success	0.09	2 months
**7**	47/M	Nil	0.09	Anterior 1/3	Positive	*M. abscessus*	Total success	0	2.5 months
**8**	27/M	Trauma	2	Anterior 2/3	Positive	*M. abscessus*	Total success	0.47	3 months
**9**	29/F	Trauma	0	Anterior 2/3	Negative	*M. abscessus*	Total success	0	2 months
**10**	67/M	Cataract surgery	2	Anterior 2/3	Positive*	*M. abscessus*	Total success	2	3 months
**11**	32/M	Foreign body	0.6	Anterior 2/3	Positive	*M. abscessus*	Total success	0.3	2 months
**12**	39/M	Cataract surgery	2.7	Full	Negative	*M. chelonae*	Failure	2.7	8 months
**13**	54/F	Cataract surgery	2	Posterior 1/3	Negative	*M. fortuitum*	Total success	2.7	1 year
**14**	40/M	Foreign body	0.17	Anterior 2/3	Positive	*M. abscessus*	Total success	0.09	2 months
**15**	24/M	Contact lens use	0.17	Anterior 2/3	Positive*	*M. abscessus*	Total success	0	4 months
**16**	73/F	Cataract surgery	2	Posterior 1/3	Negative	*M. abscessus*	Partial success	2.3	6 months
	
	

**Table 2:** Treatment of eyes infected with nontuberculous mycobacteria.


**Treatment regimen**	**% (Number) of eyes (** ***N*** ** = 12)**
Topical amikacin alone	50 (6)
Topical amikacin + clarithromycin	16.6 (2)
Topical amikacin + azithromycin	8.3 (1)
Topical amikacin + tobramycin	8.3 (1)
Topical amikacin + ciprofloxacin	8.3 (1)
Topical amikacin + azithromycin + moxifloxacin	8.3 (1)
	
	

#### PCR-based DNA sequencing targeting hsp65 gene

The amplified products underwent DNA sequencing by using an ABI prism 3110 automated DNA sequencer (Applied Biosystems, USA) followed by cycle sequencing of the amplified products in a 10-μl reaction volume, containing 1.5 μl of RR mix, 2.5 μl of sequencing buffer, 1 μl of forwarding primer/reverse primer, 4 μl MilliQ water, and 1 μl of PCR-amplified product. The Perkin–Elmer thermocycler was used for amplification using 25 cycles at 96ºC for 10 s, 50ºC for 5 s, and 60ºC for 4 min, and the initial denaturation was carried out at 96ºC for 1 min. These cycle-sequenced products were then subjected to purification and sequencing using the ABI Prism 3130 AVANT (Applied Biosystems, USA) genetic analyzer, which uses the principle of Sanger's dideoxy termination method for its working. The analysis of these sequences was done by sequence analysis software – Bio Edit sequence alignment software. Further, to confirm the sequenced data with the standard strains and establish the percentage homology, BLAST analysis (www.ncbi.nlm.nih.gov/BLAST), using PubMed, was done.

*Antibiotic susceptibility testing of NTM by disc diffusion method*

The isolated NTM were put up for AMK, CLR, AZM, Tobramycin (TOB), Ciprofloxacin (CIP), Gatifloxacin (GAT), Moxifloxacin (MOX), Ofloxacin (OFL), Norfloxacin (NOR), and Imipenem (IMP) obtained from Hi-Media Laboratories, India for determination of sensitivity pattern of the isolated NTM using Kirby Bauer disc diffusion method as per standard CLSI guidelines.

### Categorization of Patients

Patients were categorized into three groups based upon the success of medical therapy as (a) total success where complete corneal scarring occurred with no active corneal inflammation detected for at least one month post cessation of topical antibiotics; (b) partial success where additional procedures like glue and bandage contact lens was required; and (c) failure when worsening of the primary infiltrate/no response to topical antimicrobial therapy/perforation requiring therapeutic penetrating keratoplasty.

##  RESULTS

### Demographics and Clinical Details

The age of the patients at the time of presentation ranged from 24 to 73 years (average, 43.56 years). Out of a total of 16 patients, 12 were male (70.6%) and 4 female (29.4%). The time from the onset of infection to the initial presentation ranged from three weeks to eight months (median, 2.23 months). Eleven of the sixteen patients (65%) had a previous traumatic or surgical history [Table 1]. Seven out of eleven cases had trauma with vegetative or mineral matter. The four postsurgical corneal infections occurred within one year of elective cataract surgery. One patient each had a history of contact lens use and steroid use alone, respectively. In three patients, no risk factor could be identified. Out of the total infected eyes, 12 (75%) were already being treated before the presentation. Of these, six (37.5%) were on antifungals and antibiotics, five (31.3%) on antibiotics, and one (6%) on antivirals. Exposure to steroids – topical or systemic was found in 8/16 eyes (47%). The main presenting features in all patients were pain, redness, and diminution of vision. All patients presented with the stromal infiltrates at various depths associated with corneal epithelial defect [Figures 1 and 2], of which three patients had hypopyon [Table 1].

### Microbiology Results

Of the 16 patients, 10 (58.8%) tested positive for direct smear with 1% AFB stain, out of which 4 patients (40%) tested positive only on re-scraping. In all 16 patients, NTM grew on blood agar [Figure 3]. No associated bacterial or fungal infections were seen in any of the cases. The average time for culture to grow ranged from two to five days. Out of the16 isolates, 14 (87.6%) were identified as *M. abscessus*, one each (6.2%) as *M. fortuitum, M. chelonae* using PCR-based DNA sequencing targeting the heat shock protein-65 gene.

The sensitivity pattern of the 14 NTM isolates of *M. abscessus* is shown in Figure 4. The *in vitro* sensitivity showed maximum sensitivity to AMK in 14/14 eyes (100%) followed by AZM in 12/14 (85.7%) and CLR in 12/14 eyes (85.7%). The maximum resistance was to IMP, NOR (78.6%), and OFL (71.5%). The single isolate of *M. chelonae* showed sensitivity to AMK, CLR, AZM, TOB, and GAT while *M. fortuitum* NTM isolate was sensitive to AMK, CIP, MOX, GAT, and IMP.

### Clinical Course

Seventy-five percent (12/16) of eyes with culture-proven NTM infections were treated successfully with AMK alone or in combination with other antibiotics [Table 2].Fifty percent(6/12) of the eyes infected with NTM keratitis were treated with AMK alone, 25% (3/12) with a macrolide antibiotic, 8.3% (1/12) with aminoglycoside antibiotic, and 8.3% (1/12) with fluoroquinolone antibiotic. The average time taken from diagnosis of NTM till its resolution was 3.2 months (ranged from two weeks to one year).

The surgical intervention had to be done in 25% (4/16) of the infected eyes as they did not improve with the medical therapy [Table 1].Among these, one patient had impending perforation, which was managed successfully with cyanoacrylate glue and bandage contact lens, while the remaining three underwent therapeutic penetrating keratoplasty (TPK). Post TPK, the corneal button showed the same organism as identified by PCR.

The follow-up of the patients varied from two months to one year. There was no recurrence in any case. The final best-corrected visual acuity after treatment (either medical or surgical) and eradication of infection ranged from the perception of light to 6/6 [Table 1].

##  DISCUSSION

Nontuberculous mycobacteria was traditionally divided into groups I–IV by Runyon based on colony characteristics, growth rate, and specific conventional biochemical reactions.^[8]^ Groups I, II, and III included the SGM with a growth rate of two to four weeks while the rapid growers with a growth rate of seven to ten days were included in Group IV.^[6,9]^ *Mycobacterium chelonae, M. abscessus,* and *M. fortuitum* are the rapid growers which have been reported as the commonest organisms isolated from the ocular infections.^[10,11]^ Keratitis remains the most common ocular infection caused by the NTM.^[2,3,5,6]^ The prevalence of NTM infections is on the rise worldwide due to an increase in the number of refractive procedures like LASIK and endothelial keratoplasties. As per literature, the commonest NTM-causing post-LASIK keratitis was *M. chelonae* ^[12,13]^ until the most recent studies by Llovet et al^[14]^ detected an increase in infectious keratitis caused by Staphylococcus and MRSA after LASIK. Interestingly, none of our patients had a history of LASIK surgery in the past, however, four patients had cataract surgery within the last one year. All 16 patients had a unilateral presentation with male preponderance with an increased incidence in middle-aged adults matching with the available reports in the literature.^[6]^ The commonest risk factor for NTM keratitis in the present study was ocular trauma followed by surgery with delayed onset of indolent infection. Thus, NTM should be suspected as the causative agent in the delayed onset of indolent corneal ulcers.

In the present series, 75% had already received treatment in the form of antibiotics, antifungals, antivirals, and/or steroids prior to their presentation to our institute which is in accordance with the study by Girgis et al^[10]^ and Lalitha et al.^[3]^ Our patients presented with central or paracentral infiltrates at varying depths with no typical feature in contrast to a characteristic “cracked windshield” appearance described in NTM keratitis in the literature.^[11]^ In our series, 12.5% of infected eyes had ring infiltrate similar to Huanget al's^[2]^ study which may be confused with fungal or acanthamoeba keratitis. The antimicrobial susceptibility of NTM varies widely and hence the species identification of NTM is very crucial for the appropriate selection of antibiotic and better patient management. In our study, we were able to make an early diagnosis (within 24–48 hr) in 62% (10/16) as PCR-based DNA sequencing was done in all cases as opposed to earlier reports.

A similar method of rapid identification of mycobacterial species was used by Telenti et al, in 1993 targeting the same *hsp 65* region but with a different set of primers.^[7]^

The PCR-based DNA sequencing targeting *hsp65 *method is fast, cost-effective, highly specific, and efficient in comparison to the high-performance liquid chromatography (HPLC) for species identification of NTM and hence can be easily adapted by clinical microbiology laboratories with molecular microbiology facilities.^[15,16]^

Several studies have grouped the two species – *M. chelonae* and *M. abscessus* as “*M. chelonae*” because of the difficulty in separating them without molecular techniques. It is important to separate these two pathogens because they differ in their drug susceptibility.

The commonest NTM identified in our study was *M. abscessus* (87.6%; 14/16) as opposed to earlier studies, where *M. chelonae* was the predominant isolate.^[17,18]^ This higher prevalence of *M. chelonae *in earlier studies is likely a reflection of inadequate methods for NTM identification or variation among the species of NTM geographically as indicated in a study by Elliot et al.^[19]^

It has been found that *M. abscessus *carries a chromosomal erythromycin methylase gene (*erm*41) which confers inducible macrolide resistance that is not found in *M. chelonae*.^[20]^ This study showed 100% sensitivity to AMK for all NTM isolates followed by 85.7% sensitivity to macrolides (AZM and CLR) which is comparable to the previous study by Reddy et al.^[21]^ Since 87.5% were speciated to *M. abscessus* in our study, we infer that the most effective agents against *M. abscessus* are AMK, CLR, and AZM which is in agreement to a study by Elliot et al.^[19]^

Among fluoroquinolones, CIP and GAT were found to be resistant in 57.2% of *M. abscessus* isolates while MOX was resistant in 64.3% of *M. abscessus* isolates. Hence, fluoroquinolones (including the fourth generation) should not be considered among the first-line drugs for *M. abscessus *keratitis.^[19]^

Although we could not comment specifically on *M. chelonae* and *M. fortuitum* *in vitro* susceptibilities due to the low sample size (one patient in each group), we did find that *M. chelonae* had sensitivity for both aminoglycoside (TOB) and fluoroquinolone (GAT) while *M. fortuitum* showed sensitivity only to fluoroquinolones (CIP, MOX). This is in accordance with previous studies which indicate the role of fluoroquinolones in treating *M. chelonae* and *M. fortuitum* keratitis.^[18,22]^ However, studies done by Cruz et al^[23]^ and Moshirfar et al^[24]^ have shown resistance of fourth-generation fluoroquinolones to *M. chelonae*. Hence, larger sample studies with time-kill studies and minimum bactericidal concentrations (MBC) assays are required to ascertain their role.

In this study, a combination therapy was required for the clinical cure of 50% of the infected eyes. The treatment failure in some cases with the use of AMK alone might be because of the poor penetration of AMK into the intact epithelium while Kuehne et al^[25]^ reported adequate penetration of AZM and CLR into the intact corneal epithelium. On the other hand, Ford et al^[12]^ found that the topical CLR is less tolerated because of ocular discomfort and toxic reaction. Hence, based on our study sensitivity pattern, we suggest that topical AMK (2.5%) can be used either alone or in combination with oral CLR (500 mg) or AZM (500 mg) for NTM keratitis specifically for *M. abscessus*. Fluoroquinolones (including the fourth generation) remains the third line of drug for *M. abscessus* keratitis, although they have a slightly superior role for *M. chelonae* and *M. fortuitum.*

Limitations of our study include the small sample size of patients, retrospective analysis, and lack of MIC (minimum inhibitory concentration) determination for *in vitro* susceptibility of NTM.

To summarize, this study emphasizes that NTM must be kept as a differential diagnosis of infectious keratitis developing post-surgery or after injury by a foreign body injury, especially when an ulcer fails to respond adequately to the common antimicrobial drugs. It is interesting to note *M. abscessus* to be the commonest NTM-causing keratitis in contrast to the previous studies – maybe, the different geographic location plays an important role in species identification. We believe that PCR-based molecular method for definite identification of the species enables the clinicians to make an accurate and timely diagnosis. Hence molecular diagnostic testing should be included in the battery of tests when faced with such indolent ulcers. Further, therapy based on the antibiotic sensitivity pattern of NTM species helps to achieve the resolution of infection with medical therapy, thereby reducing the need for surgical intervention.

##  Financial Support and Sponsorship

Nil.

##  Conflicts of Interest

There are no conflicts of interest.
